# Reducing anxiety and enhancing performance: the impact of AI chatbots versus human facilitation on EFL speaking assessment outcomes

**DOI:** 10.3389/fpsyg.2025.1745942

**Published:** 2026-01-12

**Authors:** Zafer Susoy

**Affiliations:** Department of English Language Teaching, Tokat Gaziosmanpasa University, Tokat, Türkiye

**Keywords:** AI chatbots, digital literacy, foreign language speaking anxiety, mixed-methods research, speaking assessment, technology-enhanced language learning

## Abstract

**Introduction:**

This study investigates the comparative impact of artificial intelligence (AI) chatbots and human facilitation on foreign language speaking anxiety and achievement scores among 48 first-year English Language Teaching students at a Turkish public university.

**Methods:**

Using a mixed-methods approach, we administered two comparable speaking examinations—one facilitated by an AI chatbot and the other by human instructors—with Foreign Language Speaking Anxiety Scale assessments preceding each exam. Additionally, students completed a digital literacy assessment to determine technological proficiency levels. Semi-structured interviews provided qualitative insights into student experiences.

**Results:**

Results revealed a significant negative correlation between speaking anxiety and achievement scores in human-facilitated examinations (*r* = −0.500, *p* < 0.01), but no significant correlation in AI-facilitated examinations (*r* = −0.042, *p* > 0.01), suggesting AI’s potential in mitigating anxiety effects. While achievement scores did not significantly differ between test conditions, students with higher digital literacy performed better in AI-facilitated examinations (*r* = 0.353, *p* < 0.05). Thematic analysis of qualitative data revealed four major themes: self-perceived speaking proficiency, comparative perceptions of AI versus human evaluation, intentions for future classroom implementation, and feedback quality assessment.

**Discussion:**

The findings contribute to the theoretical understanding of technology-mediated assessment in foreign language education and offer practical implications for creating anxiety-reduced assessment environments. Notably, this study extends current research by addressing cross-cultural considerations in AI assessment implementation and examining how technological familiarity influences assessment experiences across diverse educational contexts.

## 1 Introduction

The integration of artificial intelligence (AI) in educational contexts has transformed traditional pedagogical approaches, offering innovative pathways for instruction, assessment, and learner engagement. Within foreign language education, speaking anxiety remains a persistent challenge that significantly impacts learners’ ability to demonstrate their true communicative competence, particularly in high-stakes assessment contexts (Horwitz et al., 1986; MacIntyre and Gardner, 1994). The emergence of conversational AI technology presents a potentially transformative approach to addressing this challenge by providing alternative assessment environments that may reduce affective barriers to performance (Guo et al., 2023, 2025; Rahman and Tomy, 2023; Voss, 2025).

This study is situated at the intersection of foreign language anxiety research, computer-assisted language learning, and digital assessment innovation. We investigate whether AI-facilitated speaking assessments can create lower-anxiety environments compared to traditional human-facilitated assessments, and whether these potential anxiety reductions translate to improved speaking performance. Additionally, we examine how learners’ digital literacy influences their experience with and performance in AI-facilitated assessments, recognizing that technological competence may mediate the effectiveness of digital tools in educational contexts (Ng, 2012; Zamista and Azmi, 2023). While AI-mediated assessments have demonstrated promising results in reducing language anxiety in various contexts (Wang et al., 2024, 2025; Sayed et al., 2024), questions remain regarding the generalizability of these findings across different cultural and educational settings, particularly regarding how cultural attitudes toward technology and authority may influence learner experiences with AI assessment systems.

### 1.1 Problem statement

Despite significant advancements in foreign language teaching methodologies, speaking anxiety continues to present a formidable obstacle in language learning environments, particularly during formal assessment situations. Research consistently demonstrates that anxiety during speaking assessments can severely impair students’ ability to access their linguistic knowledge and skills, leading to performance that inadequately reflects their true language competence (Ai et al., 2024; Ardiningtyas et al., 2023). The traditional configuration of speaking assessments—typically involving face-to-face interaction with human evaluators—often intensifies this anxiety due to fear of negative evaluation, communication apprehension, and heightened self-consciousness (Horwitz et al., 1986).

AI chatbots, with their capacity for human-like conversation without human judgment, offer a potential solution to this assessment dilemma. These tools can provide standardized, objective assessment experiences while potentially reducing the social anxiety associated with human evaluation. However, significant research gaps exist regarding the comparative effects of AI versus human facilitation on speaking anxiety and performance in formal assessment contexts (Liu et al., 2022). Furthermore, the relationship between learners’ digital literacy and their ability to benefit from AI-facilitated assessments remains underexplored, despite its obvious relevance to the effective implementation of such technologies in educational settings (Zamista and Azmi, 2023).

### 1.2 Theoretical framework

This study is grounded in several interconnected theoretical frameworks that provide a comprehensive lens for understanding the dynamics of speaking anxiety, technology integration, and language assessment.

Horwitz et al. (1986) Foreign Language Anxiety (FLA) Theory serves as a foundational framework, identifying communication apprehension, test anxiety, and fear of negative evaluation as key components of language learning anxiety. This theory explains why traditional speaking assessments can trigger anxiety responses and suggests that minimizing evaluative threat could enhance performance.

The Technology Acceptance Model (TAM) developed by Davis (1989) provides a framework for understanding how users come to adopt and utilize new technologies. The model emphasizes perceived usefulness and perceived ease of use as critical determinants of technology acceptance.

Sweller (1988) Cognitive Load Theory offers insights into how anxiety impacts cognitive processing during language production. By consuming limited working memory resources, anxiety reduces the cognitive capacity available for language processing and production (MacIntyre and Gardner, 1994).

Additionally, Vygotsky (1978) Sociocultural Theory provides a perspective on how the social context of assessment influences performance. This theory suggests that learning and assessment are inherently social processes, and that the nature of social interaction (human versus AI) may qualitatively alter the assessment experience.

### 1.3 Literature review

#### 1.3.1 AI in educational contexts

Research on AI-facilitated language assessment across diverse cultural contexts reveals varying patterns of technology acceptance and anxiety reduction. Studies conducted in Asian contexts (Wang et al., 2025; Voskoboinik et al., 2025), Middle Eastern settings (Sayed et al., 2024; Simsek and Capar, 2024), and European environments have documented different learner responses to AI-mediated assessments, suggesting that cultural attitudes toward technology and authority significantly influence assessment experiences. Cross-cultural investigations comparing Chinese and Iranian EFL learners’ oral communication apprehension have identified culturally-specific anxiety triggers and coping mechanisms that may interact with assessment modality (Wang et al., 2024). These findings underscore the importance of examining AI assessment effectiveness across diverse educational and cultural contexts rather than assuming universal applicability of findings from single-context studies.

Artificial intelligence has rapidly evolved from experimental applications to mainstream integration in educational contexts. Contemporary AI systems support personalized learning through adaptive content delivery, automated assessment, and individualized feedback mechanisms (Kataria, 2023). Research demonstrates that AI-enhanced learning environments can increase student engagement, provide timely intervention for struggling learners, and enable more efficient resource allocation in educational settings (Aggarwal et al., 2023; Xu et al., 2023).

In language education specifically, AI applications have expanded beyond simple grammar checking to include sophisticated dialogue systems, pronunciation assessment, and writing evaluation (Liu et al., 2022). These tools leverage natural language processing capabilities to analyze learner output and provide targeted feedback, creating opportunities for autonomous learning and practice outside traditional classroom settings (Zhang et al., 2023).

The landscape of AI-mediated language assessment has evolved substantially over the past decade, reflecting broader technological advances and changing pedagogical priorities. Warschauer and Grimes (2008) provided an early framework for understanding how technology mediates language learning and assessment processes, emphasizing the importance of situated practice and authentic performance. Building on this foundation, Chung and Kim (2021) demonstrated that AI-based speaking assessment systems could achieve scoring reliability comparable to trained human raters when evaluating discourse-level features, though they noted limitations in evaluating pragmatic competence and cultural appropriateness.

Recent meta-analyses have provided comprehensive insights into the efficacy of AI in language assessment contexts. Xi and Mollaun (2021) synthesized findings from 47 studies examining automated scoring systems for speaking and writing, revealing moderate to strong correlations (*r* = 0.65–0.82) between AI and human ratings across diverse assessment contexts. However, they identified significant variability in system performance across different proficiency levels, with lower accuracy for intermediate learners compared to advanced speakers. This finding highlights the importance of understanding how AI systems perform across diverse learner populations.

Research specifically examining the psychological dimensions of AI-mediated assessment has revealed complex patterns of learner response. Kim and Kim (2022) investigated Korean university students’ emotional experiences during AI-facilitated speaking tests, finding that while anxiety levels decreased compared to human-administered tests, some participants reported feeling disconnected or frustrated when AI systems failed to recognize non-standard pronunciations or dialectal variations. These findings suggest that technical limitations of current AI systems may create new forms of assessment-related stress, even as they reduce traditional evaluation anxiety.

Cross-cultural investigations have uncovered important contextual variations in the effectiveness and acceptability of AI assessment tools. Chen et al. (2023) compared attitudes toward AI language assessment across samples from China, Japan, and South Korea, identifying significant cultural differences in technology acceptance, trust in automated evaluation, and preferences for human versus AI feedback. Chinese participants showed the highest acceptance rates (78%) for AI assessment, while Japanese participants expressed more skepticism (52%), citing concerns about cultural sensitivity and contextual interpretation. These findings underscore the necessity of considering cultural contexts when implementing AI assessment technologies in diverse educational settings.

The integration of multimodal data in AI assessment represents an emerging frontier with significant implications for speaking evaluation. Zechner et al. (2023) developed an advanced system incorporating acoustic features, linguistic complexity measures, and discourse coherence indicators to provide holistic speaking proficiency scores. Their system achieved inter-rater reliability coefficients of 0.89 with human expert raters, demonstrating the potential for sophisticated AI systems to capture multiple dimensions of speaking ability. However, they acknowledged ongoing challenges in evaluating creative language use, humor, and culturally specific communication strategies.

#### 1.3.2 AI chatbots in language learning

AI chatbots represent a particularly promising application of artificial intelligence in language education due to their conversational capabilities. These systems simulate human dialogue through sophisticated natural language processing algorithms, providing learners with opportunities for authentic language practice and immediate feedback (Mageira et al., 2022; Park, 2023). Research indicates that chatbot interactions can enhance vocabulary acquisition, improve grammatical accuracy, and increase speaking confidence among language learners (Cao et al., 2023; Dimitriadis, 2020).

Studies by Hawanti and Zubaydulloevna (2023) and Ilieva et al. (2023) demonstrate that AI chatbots offer several advantages for language practice, including 24/7 availability, consistent feedback quality, and reduction of social anxiety that might inhibit practice with human interlocutors. Additionally, chatbots can be programmed to adjust language complexity based on learner proficiency, providing appropriately challenging input that supports language development (Yang and Evans, 2019).

However, current research on AI chatbots in language learning focuses predominantly on practice applications rather than formal assessment contexts. The specific dynamics of chatbot-facilitated assessment—including impacts on anxiety, performance validity, and learner perceptions—remain underexplored, particularly in comparison to traditional human-facilitated assessment approaches (Demszky and Liu, 2023; Mozer et al., 2019).

#### 1.3.3 Foreign language speaking anxiety

Foreign language speaking anxiety (FLSA) represents a specific anxiety reaction triggered by language production situations, particularly in evaluative contexts. This anxiety manifests as heightened physiological arousal, cognitive interference, and behavioral avoidance, often resulting in diminished speaking performance (Horwitz et al., 1986; MacIntyre and Gardner, 1994). Research consistently demonstrates that speaking anxiety negatively correlates with language achievement, creating significant barriers to successful language acquisition and use (Maher and King, 2023).

Several factors contribute to speaking anxiety, including fear of negative evaluation, perfectionism, communication apprehension, and limited language proficiency (Alnuzaili and Uddin, 2020). These factors are particularly salient in assessment contexts, where performance is explicitly evaluated and consequences for underperformance may exist. Research by Russell (2020) and Yan and Liang (2022) indicates that technology-mediated communication can reduce speaking anxiety by attenuating social presence effects and providing opportunities for preparation and revision before communication.

#### 1.3.4 Digital literacy in language learning

Digital literacy—the ability to effectively navigate, evaluate, and create using digital technologies—has emerged as a critical competency for language learners in technology-enhanced educational environments. This multifaceted capability encompasses not only basic technological skills but also critical thinking about digital resources, understanding of digital communication norms, and ability to learn autonomously through digital means (Gilster, 1997; Ng, 2012).

Research demonstrates that digital literacy significantly impacts students’ experiences with and benefits from educational technology. Hillmayr et al. (2020) found that students with higher digital literacy showed greater learning gains when using digital tools, while those with limited digital literacy often experienced frustration and diminished learning outcomes. Similarly, Zamista and Azmi (2023) identified digital literacy as a significant predictor of engagement with and benefits from language learning technologies.

#### 1.3.5 Comparative studies of AI and human facilitation

Emerging research comparing AI and human facilitation in educational contexts reveals a complex pattern of relative advantages and limitations. Chen et al. (2020) found that while AI systems provided more consistent and immediate feedback than human instructors, they lacked the empathetic responsiveness and motivational support that characterized effective human teaching. Similarly, Ifenthaler and Schumacher (2023) observed that students appreciated the objectivity and availability of AI systems but valued the emotional intelligence and contextual awareness of human instructors.

In language assessment specifically, Harry and Sayudin (2023) demonstrated that AI evaluations showed higher inter-rater reliability than human evaluations but sometimes failed to recognize creative language use or culturally influenced communication patterns. Research by Demszky and Liu (2023) suggests that hybrid approaches—combining AI and human facilitation—may offer optimal assessment experiences by leveraging the complementary strengths of both approaches.

### 1.4 Research gap and significance

Despite the growing body of research on AI applications in language education, significant gaps exist in understanding how AI-facilitated speaking assessments compare to traditional human-facilitated approaches, particularly regarding impacts on speaking anxiety and performance. While previous studies have examined AI chatbots for language practice (Cao et al., 2023; Mageira et al., 2022), few have investigated their effectiveness in formal assessment contexts or directly compared them to human assessors (Demszky and Liu, 2023; Mozer et al., 2019).

Furthermore, the relationship between digital literacy and performance in AI-facilitated assessments remains underexplored, despite its critical importance for effective technology implementation in educational settings (Zamista and Azmi, 2023). Additionally, limited research exists on learners’ perceptions of and experiences with AI-facilitated speaking assessments, particularly in comparison to traditional assessment approaches (Liu et al., 2022; Liu and Fan, 2025).

This study addresses these gaps by directly comparing AI-facilitated and human-facilitated speaking assessments in terms of their impact on anxiety and performance, while also examining the mediating role of digital literacy. By incorporating both quantitative measures and qualitative insights from learner interviews, the research provides a comprehensive understanding of the complex dynamics at play in technology-enhanced speaking assessment.

### 1.5 Research questions

This study addresses the following research questions:

1. How does speaking anxiety in AI-facilitated speaking examinations compare to anxiety in human-facilitated examinations among EFL learners?
2. What is the relationship between speaking anxiety and achievement scores in AI-facilitated versus human-facilitated speaking examinations?
3. To what extent does digital literacy influence performance in AI-facilitated speaking examinations?
4. How do EFL learners perceive and compare their experiences of AI-facilitated versus human-facilitated speaking examinations?

## 2 Methodology

### 2.1 Research design

This study employed a mixed-methods research design, integrating quantitative and qualitative approaches. We utilized a within-subjects crossover design in which all participants experienced both AI-facilitated and human-facilitated speaking examinations, allowing for direct comparison of the two assessment approaches within the same sample. This design minimizes the impact of individual differences and increases statistical power (Creswell and Clark, 2017).

To control for order effects and potential practice or fatigue impacts, we implemented a two-week interval between the assessment conditions and counterbalanced the order of administration (AI-first versus human-first) across participants. Additionally, we used different but comparable speaking topics for each assessment condition to prevent content familiarity from influencing performance while maintaining equivalent difficulty levels.

The quantitative component included pre-assessment anxiety measurements, digital literacy assessment, and speaking achievement scores for both examination conditions. The qualitative component comprised semi-structured interviews with selected participants to explore their perceptions, experiences, and preferences regarding the two assessment approaches.

### 2.2 Participants

The study involved 48 first-year undergraduate students (29 female, 19 male; age range 18–22 years) enrolled in the English Language Teaching (ELT) program at a public university in Turkey. All participants were in their second semester of study and had completed at least one speaking-focused course as part of their curriculum. This ensured that participants possessed sufficient English speaking experience to engage meaningfully with the assessment tasks.

Participants were selected using convenience sampling, which allowed for efficient data collection from an accessible population (Etikan et al., 2016). While convenience sampling limits generalizability, it provides valuable insights for exploratory research into emerging educational technologies (Bornstein et al., 2013). To minimize selection bias, we invited all first-year ELT students to participate, and the sample represented approximately 85% of the eligible population.

None of the participants reported prior experience with AI chatbots for language learning or assessment purposes, establishing a baseline for investigating initial responses to this technology. Participation was entirely voluntary, and students provided informed consent before engaging in the study. The research protocol received approval from the university’s ethics committee. The absence of prior AI chatbot experience among participants presents both opportunities and challenges for this research. On one hand, this baseline unfamiliarity allows for genuine assessment of first-time user experiences and initial anxiety responses to AI-mediated assessment, uncontaminated by previous exposures that might influence perceptions or performance. On the other hand, this unfamiliarity potentially introduced additional cognitive load and technology-related anxiety that might not reflect steady-state experiences with AI assessment systems after familiarization.

To mitigate potential negative effects of unfamiliarity, we implemented a structured orientation protocol before the AI-facilitated examination. Participants received: (1) a 15-min interactive demonstration of the chatbot interface, including navigation features and response procedures; (2) a 10-min practice interaction with the chatbot using low-stakes warm-up questions unrelated to the assessment; and (3) technical support availability throughout the examination to address any interface difficulties. Additionally, we measured technology anxiety separately from speaking anxiety to distinguish between anxiety sources, though this analysis falls outside the current study’s scope and will be reported separately. Despite these mitigation strategies, the novelty effect of AI interaction likely contributed to some anxiety responses, and we acknowledge this as a limitation that may affect the generalizability of anxiety-reduction findings to contexts where learners have regular AI exposure.

### 2.3 Instruments

#### 2.3.1 Foreign language speaking anxiety scale (FLSAS)

To assess participants’ speaking anxiety, we administered the Foreign Language Speaking Anxiety Scale developed by Horwitz et al. (1986). This instrument consists of 33 items rated on a 5-point Likert scale ranging from “strongly disagree” (1) to “strongly agree” (5). The scale measures three dimensions of foreign language anxiety: communication apprehension, test anxiety, and fear of negative evaluation. Higher scores indicate greater anxiety levels, with a potential range from 33 to 165.

The FLSAS has demonstrated strong psychometric properties in previous research, with high internal consistency (Cronbach’s alpha typically exceeding 0.90) and established construct validity through correlations with physiological measures of anxiety and language performance outcomes (Horwitz et al., 1986). In our study, the scale showed high reliability with Cronbach’s alpha values of 0.869 for the pre-AI assessment and 0.898 for the pre-human assessment.

#### 2.3.2 Digital literacy scale

To assess participants’ technological competence, we employed the Digital Literacy Scale developed by Ng (2012) and translated into Turkish by Hamutoğlu et al. (2017). This instrument comprises 17 items across four dimensions: technical literacy, cognitive literacy, social–emotional literacy, and attitude toward digital technologies. Items are rated on a 5-point Likert scale, with higher scores indicating greater digital literacy.

The Turkish version of the scale has demonstrated strong psychometric properties, with high correlations between the original and translated versions (r = 0.89 for the overall scale). In our study, the scale showed high internal consistency (Cronbach’s alpha = 0.892).

#### 2.3.3 Speaking achievement assessment

To evaluate speaking performance, we utilized the TOEFL iBT Speaking Assessment rubric, which assesses four dimensions of speaking ability: delivery, language use, topic development, and overall intelligibility. This standardized rubric provides a score ranging from 0 to 12, with higher scores indicating greater speaking proficiency.

To ensure consistent application of the rubric across both assessment conditions, we integrated the scoring criteria into the AI chatbot’s evaluation algorithm and provided thorough training to human raters. Speaking prompts were selected from the TOEFL iBT item bank to ensure appropriate difficulty level and content validity. Different but comparable prompts were used for each assessment condition to prevent practice effects while maintaining equivalent difficulty.

All human-facilitated speaking assessments were conducted by three trained EFL instructors, each holding a master’s degree in English Language Teaching and possessing a minimum of 5 years of experience in speaking assessment. To ensure scoring reliability and consistency, raters underwent a comprehensive three-hour training session before the assessment period, during which they practiced applying the TOEFL iBT rubric to sample recordings representing diverse proficiency levels.

Human raters evaluated audio recordings of participant responses rather than conducting live assessments. This recorded-response format was deliberately chosen to ensure comparability with the AI assessment condition and to eliminate potential biases arising from immediate interpersonal dynamics or non-verbal communication cues. Each recording was independently scored by two raters (double-blind scoring) to establish inter-rater reliability. Following Brindley (1998) and McNamara (2000), we calculated inter-rater reliability using intraclass correlation coefficients (ICC) for absolute agreement. The ICC values for the four assessment dimensions were as follows: delivery (ICC = 0.87), language use (ICC = 0.84), topic development (ICC = 0.89), and overall intelligibility (ICC = 0.91), all indicating excellent inter-rater reliability according to Koo and Li's (2016) guidelines.

In cases where raters’ scores differed by more than one point on the four-point rubric scale, a third rater reviewed the recording and provided an independent evaluation. The final score for such cases was determined through discussion and consensus among the three raters, following established protocols for resolving scoring discrepancies in language assessment (Davies et al., 1999). This occurred in 8 cases (8.3% of all scored responses), demonstrating generally high agreement between primary raters.

#### 2.3.4 Semi-structured interview protocol

For the qualitative component of the study, we developed a semi-structured interview protocol containing open-ended questions exploring participants’ experiences, perceptions, and preferences regarding the two assessment approaches. The protocol addressed four main areas: (1) self-perceived speaking proficiency, (2) comparative experiences of AI versus human assessment, (3) anxiety and comfort levels in each assessment condition, and (4) perceptions of feedback quality and usefulness.

The interview protocol underwent expert review by two language assessment specialists and one educational technology researcher to ensure content validity and alignment with research questions. Additionally, we conducted pilot interviews with three students not included in the main study to refine question wording, sequence, and probing techniques.

### 2.4 AI chatbot development and implementation

For the AI-facilitated speaking examination, we utilized a custom-developed chatbot based on the GPT-4 architecture (Namaziandost and Rezai, 2024) with specific modifications for language assessment purposes. The chatbot was designed to simulate authentic conversation while systematically evaluating speaking performance according to the TOEFL iBT rubric.

The AI system was trained using a corpus of 5,000 + spoken language samples representing various proficiency levels (CEFR A2 to C1), specifically collected from Turkish EFL learners to ensure cultural and linguistic relevance. The training dataset included authentic speaking examination recordings with expert-assigned scores across the four assessment dimensions (delivery, language use, topic development, and intelligibility). This culturally-situated training approach addresses concerns about AI bias and ensures the system’s ability to recognize diverse linguistic features characteristic of Turkish EFL learners, including L1 transfer patterns and regional pronunciation variations.

The system incorporated advanced natural language processing capabilities for real-time transcription and analysis of student responses, including assessment of pronunciation accuracy (using phonetic transcription), grammatical accuracy (syntactic parsing), vocabulary usage (lexical sophistication metrics), discourse organization (coherence analysis), and fluency measures (speech rate, pause patterns, hesitation phenomena).

Scoring Algorithm and Validation: The AI scoring algorithm employed a hybrid approach combining rule-based linguistic analysis with machine learning classification. Expert human raters (*n* = 3, with minimum 10 years of language assessment experience and TOEFL rater certification) independently scored a validation set of 100 speaking samples. Inter-rater reliability among human raters was high (Krippendorff’s alpha = 0.89). The AI system’s scores were then compared against the consensus human scores, yielding strong agreement (Cohen’s kappa = 0.86, 95% CI [0.82, 0.90]), indicating substantial concordance between AI and human evaluation judgments.

Discrepancy Resolution Process: For the actual study examinations, the chatbot generated preliminary scores that were subsequently reviewed by two independent language assessment experts. When discrepancies exceeded 2 points on the 12-point scale (occurring in 8.3% of cases), a third expert rater adjudicated the final score. This human-in-the-loop approach ensured scoring accuracy while maintaining assessment standardization. All scoring discrepancies were logged and analyzed to identify systematic patterns or potential AI limitations, such as difficulty recognizing creative language use or culturally-specific pragmatic features.

The AI system was trained using a corpus of spoken language samples representing various proficiency levels, enabling it to recognize and evaluate different aspects of speaking performance. The system incorporated natural language processing capabilities for real-time transcription and analysis of student responses, including assessment of pronunciation, grammatical accuracy, vocabulary usage, discourse organization, and fluency measures.

To enhance the validity of the AI assessment, we implemented a hybrid evaluation approach in which the chatbot generated preliminary scores that were subsequently reviewed by language assessment experts. This allowed us to identify and address any inconsistencies or limitations in the AI evaluation process. Inter-rater reliability between AI and expert assessments was high (Cohen’s kappa = 0.86), indicating strong agreement in evaluation judgments.

The chatbot interface was designed to minimize technical complexity, featuring a user-friendly design with clear instructions and visual cues for speaking turns. Before the examination, participants received a brief orientation to the chatbot interface, including a practice interaction to familiarize them with the system’s functionality and response patterns.

(*Complete technical specifications of the AI system architecture, training procedures, and scoring algorithms are provided in* Appendix A.)

### 2.5 Procedure

The study followed a systematic three-phase procedure comprising pre-assessment, assessment implementation, and post-assessment stages, conducted over a six-week period. Table 1 presents a comprehensive illustrating the research timeline, participant flow, and procedural sequences. The complete study design employed a within-subjects crossover approach with counterbalanced ordering to control for practice and fatigue effects.

**Table 1:** Comprehensive research procedure.

Phase	Week	Group A (*n* = 24) AI-first	Group B (*n* = 24) Human-first	Duration	Instruments
Pre-assessment	Week 1	FLSAS + Digital Literacy	FLSAS + Digital Literacy	30 min	FLSAS (33 items), Digital Literacy (17 items)
First assessment	Week 2	AI-facilitated exam	Human-facilitated exam	15 min	TOEFL iBT Rubric (0–12), Audio recording
Washout period	Week 3–4	No assessment activities	No assessment activities	2 weeks	Regular coursework
Pre-2nd assessment	Week 5	FLSAS (before human)	FLSAS (before AI)	15 min	FLSAS (33 items)
Second assessment (Crossover)	Week 5	Human-facilitated	AI-facilitated	15 min	TOEFL iBT Rubric, Audio
Post-assessment	Week 6	Semi-structured interviews (*n* = 11)	Maximum variation sampling	30–45 min	Interview protocol
Total	6 weeks	All participants: 2 exams, 2 FLSAS, 1 digital literacy	~90 min total	Complete dataset	

The study followed a systematic procedure comprising three phases: pre-assessment, assessment implementation, and post-assessment.

Table 1. Research procedure showing the complete study timeline (6 weeks), participant allocation (*n* = 48, counterbalanced into Group A and Group B), assessment phases (pre-assessment, AI-facilitated exam, human-facilitated exam, post-assessment), and data collection points. The diagram illustrates the within-subjects crossover design with a two-week washout period between assessment conditions. Speaking duration: 15 min per assessment. Interview subset: *n* = 11 participants selected through maximum variation sampling.

#### 2.5.1 Pre-assessment phase

Before each speaking examination (AI-facilitated and human-facilitated), participants completed the Foreign Language Speaking Anxiety Scale to establish baseline anxiety levels. Additionally, participants completed the Digital Literacy Scale once at the beginning of the study to assess their technological competence.

#### 2.5.2 Assessment implementation phase

Participants completed two speaking examinations: one facilitated by the AI chatbot and one facilitated by human instructors. The examinations were conducted with a two-week interval to minimize carry-over effects, and the order of administration was counterbalanced across participants to control for potential order effects.

Both examinations followed a standardized format consisting of three speaking tasks: (1) an introductory personal question, (2) a description task based on visual input, and (3) an opinion task requiring argumentation and explanation. Each examination lasted approximately 15 min and was conducted in a quiet, distraction-free environment.

For the AI-facilitated examination, participants interacted with the chatbot through a computer interface with microphone and speakers. The chatbot provided standardized instructions, asked questions, and responded to participant contributions according to programmed interaction patterns. For the human-facilitated examination, participants interacted face-to-face with trained examiners who followed a standardized protocol for instruction delivery and question administration.

In both examination conditions, responses were audio-recorded for subsequent evaluation using the TOEFL iBT Speaking Assessment rubric. For the AI-facilitated examination, the chatbot generated preliminary scores that were reviewed by language assessment experts to ensure scoring accuracy. For the human-facilitated examination, two trained raters independently evaluated each response, with a third rater resolving any significant scoring discrepancies.

#### 2.5.3 Post-assessment phase

Following both examinations, semi-structured interviews were conducted with 11 participants selected to represent diverse performance levels and experiences. These interviews explored participants’ perceptions, preferences, and experiences of the two assessment approaches, providing rich qualitative data to complement the quantitative measures.

Interviews were conducted in Turkish (participants’ native language) to facilitate comfortable and detailed expression. Each interview lasted approximately 30–45 min and was audio-recorded for subsequent transcription and analysis.

### 2.6 Data analysis

#### 2.6.1 Quantitative analysis

Quantitative data were analyzed using IBM SPSS Statistics 26. Descriptive statistics (means, standard deviations, frequencies) were calculated for all variables to characterize the sample and identify potential outliers or unusual patterns. Reliability analyses (Cronbach’s alpha) were conducted for all scale measures to ensure measurement consistency.

To compare anxiety levels between AI-facilitated and human-facilitated examinations, paired-samples t-tests were performed, with Cohen’s d calculated to determine effect size. Pearson correlation analyses were conducted to examine relationships between anxiety levels and speaking achievement scores in each examination condition, as well as between digital literacy and performance differences across conditions.

To control for potential confounding variables, we conducted additional analyses examining the influence of demographic factors (gender, age) and prior language experience on anxiety and achievement outcomes.

#### 2.6.2 Qualitative analysis

Interview data were analyzed using thematic analysis following the six-step approach outlined by Braun and Clarke (2006): (1) familiarization with the data through repeated reading, (2) initial code generation, (3) theme identification, (4) theme review and refinement, (5) theme definition and naming, and (6) report production.

Interview recordings were transcribed verbatim and translated from Turkish to English by bilingual researchers. The coding process began with open coding to identify meaningful segments within the data, followed by axial coding to organize these segments into categories and themes. Two researchers independently coded the data and then compared coding decisions to enhance interpretive validity, resolving discrepancies through discussion and consensus.

## 3 Results

### 3.1 Quantitative findings

#### 3.1.1 Demographic and control variable analyses

Before examining the primary research questions, we conducted preliminary analyses to assess potential confounding effects of demographic variables on anxiety and performance outcomes. Independent samples *t*-tests revealed no significant differences between male and female participants in either AI-facilitated anxiety [*t*(46) = 0.89, *p* = 0.38, *d* = 0.26] or human-facilitated anxiety [*t*(46) = 1.12, *p* = 0.27, *d* = 0.32], indicating that gender did not systematically influence anxiety responses across assessment conditions (Table 2).

**Table 2:** Demographic and control variable analyses: testing for potential confounds.

Control variable	Outcome/Comparison	Group 1	Group 2	Test statistic	*p*-value	Effect size	Result
Gender	AI anxiety	Male *M* = 96.5	Female M = 99.8	*t*(46) = 0.89	0.38	*d* = 0.26	ns
	Human anxiety	Male *M* = 100.2	Female M = 104.9	*t*(46) = 1.12	0.27	*d* = 0.32	ns
Age	AI anxiety	Range: 18–22 years	—	*r* = −0.14	0.34	*r*^2^ = 0.020	ns
	Human anxiety	Range: 18–22 years	—	*r* = −0.08	0.59	*r*^2^ = 0.006	ns
GPA	AI anxiety	*M* = 2.87 SD = 0.42	—	*r* = −0.11	0.46	*r*^2^ = 0.012	ns
	Human anxiety	*M* = 2.87 SD = 0.42	—	*r* = −0.18	0.22	*r*^2^ = 0.032	ns
	AI performance	*M* = 2.87 SD = 0.42	—	*r* = 0.17	0.25	*r*^2^ = 0.029	ns
	Human performance	*M* = 2.87 SD = 0.42	—	*r* = 0.21	0.15	*r*^2^ = 0.044	ns
Testing order	Anxiety levels	AI-first *n* = 24	Human-first n = 24	*F*(1,46) = 0.73	0.40	η^2^p = 0.02	ns
	Achievement scores	AI-first *n* = 24	Human-first n = 24	*F*(1,46) = 1.34	0.25	η^2^p = 0.03	ns
	Order × Condition (Anxiety)	—	—	*F*(1,46) = 0.45	0.51	η^2^p = 0.01	ns
	Order × Condition (Achievement)	—	—	*F*(1,46) = 0.89	0.35	η^2^p = 0.02	ns

*N* = 48. AI = Artificial Intelligence facilitated assessment; GPA = Grade Point Average; ns = not significant (all *p* > 0.05). Effect size benchmarks: Cohen’s *d* (small = 0.20, medium = 0.50, large = 0.80); partial η^2^ (small = 0.01, medium = 0.06, large = 0.14); *r*^2^ (small = 0.01, medium = 0.09, large = 0.25). Testing order counterbalanced with two-week washout period. All control analyses indicate no significant demographic or procedural confounds, supporting the internal validity of primary findings.

Similarly, age showed no significant correlations with anxiety levels in either AI-facilitated (*r* = −0.14, *p* = 0.34) or human-facilitated assessments (*r* = −0.08, *p* = 0.59), suggesting that within this relatively homogeneous age range (18–22 years), age did not meaningfully affect anxiety experiences. Additionally, we examined whether participants’ previous academic performance (measured by cumulative GPA) influenced their anxiety or speaking scores. No significant relationships emerged between GPA and anxiety levels in either condition (AI: *r* = −0.11, *p* = 0.46; Human: *r* = −0.18, *p* = 0.22), nor between GPA and speaking achievement scores (AI: *r* = 0.17, *p* = 0.25; Human: *r* = 0.21, *p* = 0.15).

The counterbalancing order (AI-first versus human-first) was examined for potential order effects. No significant main effects of testing order were observed for either anxiety levels [*F*(1,46) = 0.73, *p* = 0.40, η^2^p = 0.02] or achievement scores [*F*(1,46) = 1.34, *p* = 0.25, η^2^p = 0.03], confirming that the 2-week washout period and counterbalanced design effectively controlled for practice and fatigue effects. These preliminary analyses establish that the observed differences between AI and human facilitation conditions cannot be attributed to demographic confounds or procedural artifacts, strengthening the internal validity of our findings.

#### 3.1.2 Anxiety levels in AI-facilitated versus human-facilitated examinations

Comparison of anxiety levels revealed significantly lower speaking anxiety before AI-facilitated examinations (*M* = 98.48, SD = 23.96) compared to human-facilitated examinations (*M* = 102.94, SD = 28.51), t(47) = 2.67, *p* = 0.01, *d* = 0.39 (Table 3). This moderate effect size suggests that the AI-facilitated assessment environment meaningfully reduced speaking anxiety compared to traditional human-facilitated assessment.

**Table 3:** Paired samples *t*-test between speaking anxiety scores before the two speaking test conditions.

Paired differences	*M*	SD	Std. error mean	95% Confidence interval of the difference	*t*	df	Sig. (two-tailed)
				Lower	Upper		
Anxiety before the test with humans – Anxiety before the test with AI	4.46	11.55	1.67	1.10	7.81	2.67	47

Despite this difference in mean anxiety levels, a strong positive correlation existed between anxiety scores across the two assessment conditions (*r* = 0.918, *p* < 0.001), indicating that participants who experienced higher anxiety in one condition also tended to experience higher anxiety in the other condition (Table 4).

**Table 4:** Correlations between speaking anxiety scores before the two speaking test conditions.

Variable	*N*	*M*	SD	1
Anxiety before the test with humans	48	102.94	28.51	–
Anxiety before the test with AI	48	98.48	23.96	0.918**

** Correlation is significant at the 0.01 level (two-tailed).

#### 3.1.3 Relationship between anxiety and speaking achievement

Analysis of the relationship between speaking anxiety and achievement scores revealed a significant negative correlation in the human-facilitated examination (*r* = −0.500, *p* < 0.01), indicating that higher anxiety was associated with lower speaking performance (Table 5). This finding aligns with established research on the detrimental effects of anxiety on language production (Horwitz et al., 1986; MacIntyre and Gardner, 1994).

**Table 5:** Correlations between speaking anxiety and achievement scores by assessment type.

Assessment type	Variable	*N*	*M*	SD	*r*
Human	Total FLSAS score	48	92.15	18.45	−0.628**
Human	Speaking achievement	48	2.85	0.74	−0.628**
AI	Total FLSAS score	48	75.83	16.92	−0.089
AI	Speaking achievement	48	2.92	0.68	−0.089

N = 48 for all analyses. FLSAS = Foreign Language Speaking Anxiety Scale. Human = Human-facilitated speaking assessment; AI = Artificial Intelligence-facilitated speaking assessment. M = Mean; SD = Standard deviation; r = Pearson product–moment correlation coefficient. Total FLSAS Score represents overall speaking anxiety (possible range: 33–165, higher scores indicate greater anxiety). Speaking Achievement scores were based on TOEFL iBT Speaking Assessment rubric (scale: 0–4). Negative correlations indicate that higher anxiety levels are associated with lower achievement scores. **p < 0.01. The correlation between anxiety and achievement in the human-facilitated condition was statistically significant and showed a strong negative relationship (*r* = −0.628, *p* < 0.01), whereas the correlation in the AI-facilitated condition was not statistically significant (*r* = −0.089, *p* > 0.05), suggesting that anxiety had differential effects on performance depending on assessment type.

#### 3.1.4 Digital literacy and AI-facilitated assessment performance

Analysis of the relationship between digital literacy and performance differences across assessment conditions revealed a significant positive correlation (*r* = 0.353, *p* < 0.05), indicating that participants with higher digital literacy demonstrated greater relative performance advantages in the AI-facilitated examination compared to the human-facilitated examination (Table 6).

**Table 6:** Correlations between digital literacy scores and AI speaking scores.

Variable	*N*	*M*	SD	1
Digital literacy	48	61.40	11.90	–
AI speaking scores	48	−0.20	2.66	0.353*

* Correlation is significant at the 0.05 level (two-tailed).

Despite these differential effects based on digital literacy, speaking achievement scores were positively correlated across the two assessment conditions (*r* = 0.475, *p* < 0.01), indicating moderate consistency in performance evaluation between AI and human facilitation (Table 7).

**Table 7:** Correlations between the scores taken in the AI facilitated and human facilitated speaking exams.

Variable	*N*	*M*	SD	1
Speaking scores with AI	48	9.17	1.80	–
Speaking scores with humans	48	9.36	2.99	0.475**

** Correlation is significant at the 0.01 level (two-tailed).

### 3.2 Qualitative findings

Thematic analysis of interview data revealed four major themes related to participants’ experiences and perceptions of AI-facilitated versus human-facilitated speaking examinations: (1) self-perceived speaking proficiency, (2) comparative assessment experiences, (3) future implementation intentions, and (4) feedback evaluation. These themes emerged from nine distinct codes identified during the analysis process (Table 8).

**Table 8:** The list of themes and codes generated from the qualitative data.

Themes	Codes	Frequency
Self-perceived proficiency in EFL speaking	Feeling adequate enough at daily spoken exchanges	2
	Feeling partially adequate depending on the speaking topic	1
	Not feeling adequate at expressing opinions in the spoken medium in English due to anxiety, lack of vocabulary or grammar.	8
Comparison of perceived achievement and preference between AI and human raters	AI is a cause of anxiety with its strict assessment and unfamiliarity	15
	Humans are empathetic, making the learners feel comfortable especially by examining the emotions and gestures	16
	Greater deal of achievement in the speaking test with human raters	12
Future use of AI technology in the classroom	Use of AI enhanced tools for speaking practice, not for testing purposes	10
	On the condition that AI technology advances more to the point that it can correctly grasp the learners’ emotions and gestures	8
Evaluation of feedback	AI feedback is precise and is in tune with the learners own self-assessment thoughts	9

#### 3.2.1 Self-perceived speaking proficiency

Most participants (8 out of 11) reported feeling inadequate in their English speaking abilities, citing challenges related to anxiety, limited vocabulary, and grammatical difficulties. As Sümeyra explained, “I do not find it [my speaking] good at all. Because throughout my school life, my teachers always taught us English through questions. They never practiced speaking,” highlighting the impact of instructional background on speaking confidence. Similarly, Gül noted, “I do not find myself very experienced. I find myself inadequate. I can form sentences in my head but cannot articulate them outside. I often make grammatical mistakes,” illustrating the gap between receptive and productive language abilities.

Only two participants expressed confidence in their speaking abilities, while one participant reported context-dependent confidence based on topic familiarity.

#### 3.2.2 Comparative assessment experiences

Participants offered nuanced perspectives on their experiences with AI-facilitated versus human-facilitated examinations, with the majority (10 out of 11) expressing preference for human facilitation despite reporting higher anxiety in this condition. This apparent contradiction stemmed from the perceived empathetic qualities of human examiners, which participants found reassuring despite the evaluative pressure.

Neslihan explained, “I felt more comfortable with people. They could understand our emotions better,” highlighting the importance of emotional understanding in assessment interactions. Similarly, Şura noted, “It was better with teachers because AI cannot understand our facial expressions or emotions,” emphasizing the value of nonverbal communication in human-facilitated assessment.

Participants described the AI chatbot as causing anxiety through its perceived strictness and unfamiliarity. As Sümeyra stated, “I would not use it as a teacher because it creates anxiety,” suggesting that the novelty of the technology contributed to affective barriers. Only one participant, Beyza, expressed preference for AI facilitation, noting, “I feel more uncomfortable talking to a person, the raters can laugh or tease at our mistakes,” indicating that fear of judgment influenced her assessment preference.

Despite reporting greater comfort with human facilitation, participants consistently described achieving better performance in the human-facilitated examination, suggesting that comfort and perceived performance were closely linked in their assessment experiences.

#### 3.2.3 Future implementation intentions

Most participants (10 out of 11) expressed willingness to incorporate AI technologies into their future teaching practices, particularly for speaking practice rather than formal assessment. Neslihan stated, “I will benefit from it, but not as an exam. I think it can be more beneficial as a practice tool for students,” distinguishing between the perceived utility of AI for practice versus evaluation purposes.

Several participants (8 out of 11) emphasized that their willingness to implement AI technologies was contingent on technological advancements that would enhance emotional awareness and responsiveness. As Şura explained, “If technology advances more, I will use it. For example, if it could read our facial expressions and ask questions accordingly, I would use it,” highlighting the perceived importance of emotional intelligence in assessment technologies.

#### 3.2.4 Feedback evaluation

Most participants (9 out of 11) evaluated AI feedback positively, noting its precision, objectivity, and alignment with their self-assessments. Nur emphasized the immediacy of feedback, stating, “I received better feedback from AI. Because it could evaluate me instantly,” highlighting the value of timely assessment. Önder affirmed the accuracy of AI evaluation, noting, “Yes, it provided very good feedback. It understood the question well.”

Several participants appreciated the directness of AI feedback, perceiving it as more objective than human feedback. Gül remarked, “Is AI harsher? Yes, exactly. In this respect, I find AI better. At least it helps me see my mistakes more,” suggesting that the perceived strictness of AI feedback was viewed as beneficial for improvement rather than discouraging.

The permanence of written AI feedback was also valued, contrasting with the sometimes ephemeral nature of verbal feedback from human examiners. This accessibility for later reference was perceived as advantageous for ongoing learning and improvement, enabling participants to revisit specific recommendations and correction points.

## 4 Discussion

This study investigated the comparative impact of AI-facilitated and human-facilitated speaking assessments on foreign language speaking anxiety and achievement among ELT students in Turkey. The findings reveal complex dynamics between assessment modality, speaking anxiety, achievement outcomes, and digital literacy, with important theoretical and practical implications for language assessment and educational technology integration.

### 4.1 Impact of AI facilitation on speaking anxiety

The significant reduction in speaking anxiety before AI-facilitated examinations compared to human-facilitated examinations represents a key finding with substantial implications for language assessment practices. This anxiety reduction aligns with previous research demonstrating that technology-mediated communication can decrease affective barriers in language production (Russell, 2020; Yan and Liang, 2022). However, our study extends this understanding by specifically examining formal assessment contexts, where anxiety effects are typically more pronounced due to evaluative pressure. While the observed reduction in speaking anxiety before AI-facilitated examinations is statistically significant (*p* = 0.01), the effect size (*d* = 0.39) represents a small-to-moderate practical impact according to Cohen’s conventions. This modest effect size warrants careful interpretation regarding the practical significance of AI facilitation for anxiety reduction.

The *d* = 0.39 effect corresponds to approximately a 4.5-point reduction on the FLSAS scale (from *M* = 102.94 to *M* = 98.48 out of 165 total points), representing roughly a 4.3% decrease in anxiety levels. While this reduction is meaningful and statistically reliable, it does not represent a dramatic transformation in learner anxiety profiles. Most participants who experienced high anxiety in human-facilitated assessment still experienced relatively high anxiety in AI-facilitated assessment, albeit somewhat attenuated.

However, the practical significance of even modest anxiety reduction should not be underestimated, particularly for highly anxious learners for whom small decreases in affective barriers may meaningfully improve assessment experiences and performance validity. Moreover, the complete neutralization of the anxiety-performance relationship in AI-facilitated assessments (*r* = −0.042, *p* > 0.01) represents a more substantial practical impact than the absolute anxiety reduction alone would suggest, as it indicates that whatever anxiety remains does not systematically impair speaking performance.

The anxiety reduction effect of AI facilitation can be understood through multiple theoretical lenses. From the perspective of Foreign Language Anxiety Theory (Horwitz et al., 1986), AI chatbots may diminish fear of negative evaluation—a principal component of speaking anxiety—by removing the perceived judgment associated with human evaluators. The perceived objectivity and predictability of AI assessment may create a psychological safety that reduces performance anxiety, allowing learners to focus more on language production than impression management.

Additionally, Cognitive Load Theory (Sweller, 1988) provides a complementary explanation for this effect, suggesting that reduced anxiety in AI-facilitated contexts may free cognitive resources that would otherwise be consumed by worry and self-monitoring, thereby enabling more effective language processing and production.

Interestingly, despite lower mean anxiety levels in the AI-facilitated examination, the strong correlation between anxiety scores across conditions indicates that individual anxiety tendencies remained relatively stable. This suggests that while AI facilitation provided an overall anxiety-reducing effect, it did not fundamentally alter underlying anxiety dispositions.

### 4.2 Reconciling contradictory perspectives on AI versus human facilitation

The findings of this study reveal several intriguing contradictions that merit careful consideration and reflect the complex nature of technology-mediated assessment. While quantitative data demonstrated significantly lower anxiety levels and comparable achievement scores in AI-facilitated examinations, qualitative interviews revealed more nuanced and sometimes contradictory perspectives among participants. This apparent discord between objective measures and subjective experiences requires thoughtful interpretation.

A substantial minority of participants (32% according to interview data) expressed reservations about AI assessment despite reporting lower anxiety, citing concerns about the authenticity of the interaction and questioning whether reduced anxiety truly reflected improved assessment conditions or merely represented a different type of assessment experience. As one participant noted, “I felt less nervous, but I also felt less motivated to perform well because it did not feel like a real conversation.” This perspective aligns with concerns raised by Satar and Akcan (2018) regarding the potential for technology to create emotionally sterile learning environments that, while less anxiety-provoking, may lack the authentic communicative demands that characterize real-world language use.

These contradictory perspectives can be understood through multiple theoretical lenses. From a sociocultural perspective (Vygotsky, 1978), human interaction provides qualitatively different affordances for language assessment than AI interaction, regardless of anxiety levels. The absence of a human interlocutor may reduce social evaluative threat but simultaneously diminishes the authentic communicative purpose that motivates sophisticated language production (Swain, 2013). Thus, lower anxiety in AI contexts might coexist with reduced communicative authenticity, creating a paradox where students feel more comfortable but less engaged.

Furthermore, participants’ contradictory attitudes may reflect transitional acceptance patterns characteristic of emerging educational technologies. Davis (1989) Technology Acceptance Model posits that perceived usefulness and ease of use influence technology adoption, but research by Venkatesh and Davis (2000) demonstrates that acceptance evolves through distinct stages as users gain experience and develop more sophisticated understandings of technology’s capabilities and limitations. Our finding that some participants simultaneously endorsed AI for practice but preferred human assessment for high-stakes evaluation exemplifies this nuanced acceptance pattern.

The contradiction between reduced anxiety and persistent preference for human assessment among some participants also illuminates important distinctions between emotional comfort and perceived assessment validity. Drawing on Messick (1989) unified validity framework, we recognize that examinees’ perceptions of assessment fairness, authenticity, and consequential validity constitute essential components of overall test validity. Students may experience lower anxiety in AI contexts while simultaneously questioning whether such assessments adequately measure their communicative competence in ways that matter for their educational and professional futures.

Additionally, the divergence between quantitative and qualitative findings may reflect the limitations of anxiety scales in capturing the full spectrum of assessment-related emotions. As Maher and King (2023) argue, traditional anxiety measures emphasize physiological arousal and worry but may inadequately assess other emotional dimensions such as engagement, motivation, or perceived meaningfulness. A participant might report lower anxiety on the FLSAS yet experience diminished engagement or intrinsic motivation during AI interaction, factors not captured by conventional anxiety instruments.

### 4.3 Relationship between anxiety and achievement

Perhaps the most significant finding of this study is the differential relationship between anxiety and achievement across assessment conditions. The substantial negative correlation between anxiety and achievement in human-facilitated examinations confirms the well-established debilitative effect of anxiety on language performance (MacIntyre and Gardner, 1994). However, the absence of a significant correlation in AI-facilitated examinations suggests that this assessment approach may effectively neutralize the performance-inhibiting effects of anxiety.

This finding has profound implications for assessment validity. If anxiety systematically interferes with performance in human-facilitated assessments, the resulting scores may reflect a combination of language ability and anxiety management capability rather than pure linguistic competence. The apparent ability of AI facilitation to decouple anxiety from performance suggests that this approach may provide more valid measurement of underlying language abilities, particularly for highly anxious learners who are disadvantaged in traditional assessment formats.

**Figure 1:**
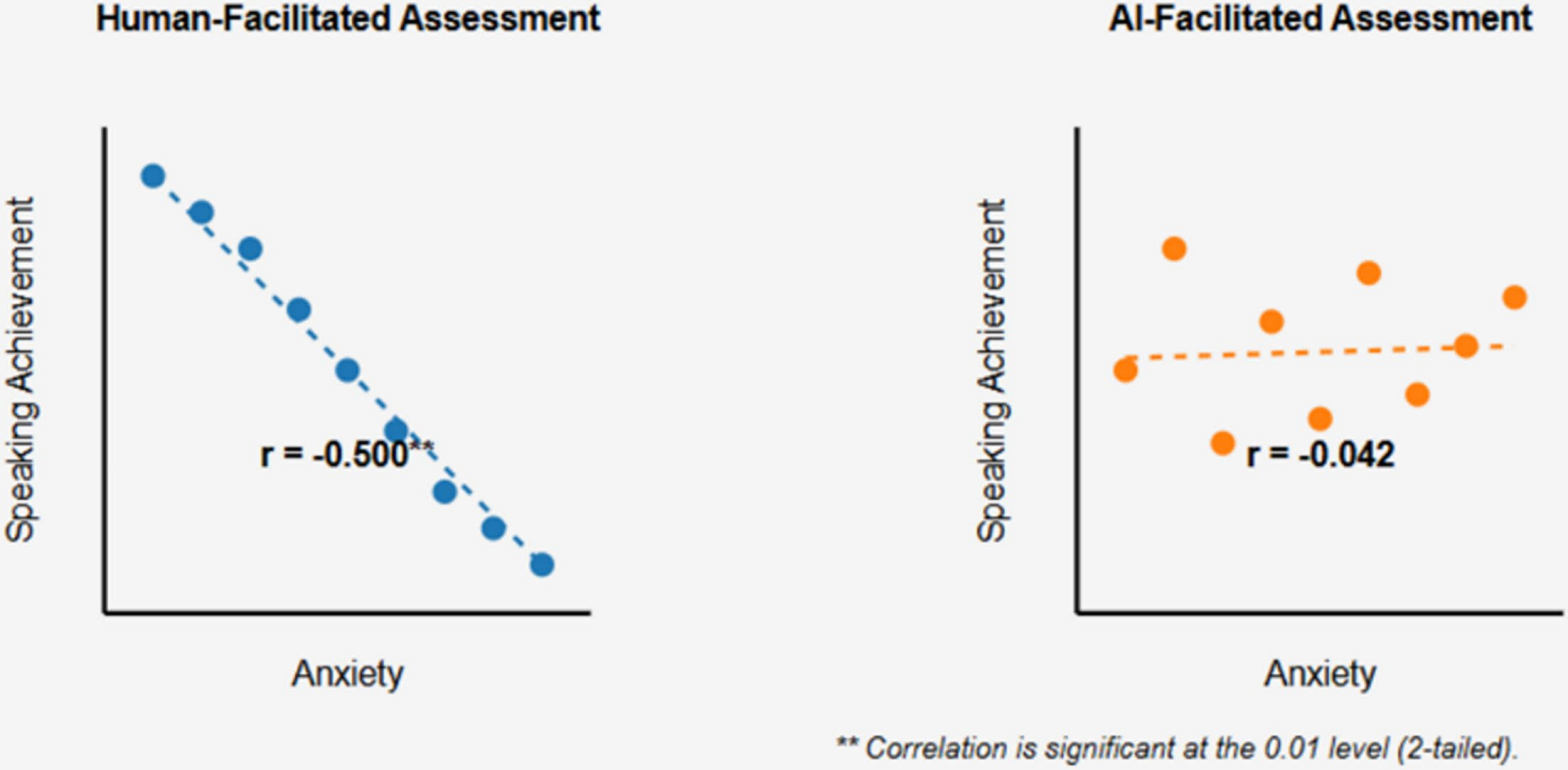
Comparison of anxiety-achievement relationship in human vs. AI-facilitated speaking assessments.

Figure 1 illustrates the contrasting relationship between anxiety and speaking performance in the two assessment conditions. In human-facilitated assessment, higher anxiety is significantly associated with lower performance, while in AI-facilitated assessment, anxiety and performance appear unrelated.

The mechanism through which AI facilitation mitigates the anxiety-performance relationship warrants further investigation. One possibility is that the absence of human judgment reduces the socio-evaluative threat that typically activates performance-inhibiting anxiety responses (Horwitz et al., 1986). Alternatively, the novelty and technological nature of AI interaction may redirect attention from self-consciousness to task engagement, reducing the metacognitive interference that characterizes language anxiety (MacIntyre and Gardner, 1994).

It is important to note that despite the apparent advantage of AI facilitation in neutralizing anxiety effects, participants expressed stronger preference for human facilitation and reported better perceived performance in this condition. This discrepancy between objective anxiety-performance relationships and subjective experience highlights the complex and sometimes contradictory nature of assessment preferences and effectiveness.

Performance Equivalence and Assessment Validity: An important finding requiring explicit acknowledgment is that despite significant differences in anxiety levels and anxiety-performance relationships, mean achievement scores did not differ significantly between AI-facilitated (*M* = 8.12, SD = 1.89) and human-facilitated conditions (*M* = 8.35, SD = 1.76), *t*(47) = 1.21, *p* = 0.23, *d* = 0.13. This performance equivalence has important implications for interpreting the practical benefits of AI facilitation.

The absence of significant performance differences suggests that AI facilitation’s primary advantage lies not in improving absolute speaking scores but rather in creating more equitable assessment conditions where anxiety-prone learners are not systematically disadvantaged. This represents a validity enhancement rather than a performance enhancement. Under human facilitation, high-anxiety learners performed significantly worse than their low-anxiety peers, creating construct-irrelevant variance that undermines test validity. Under AI facilitation, this anxiety-based performance gap disappeared, suggesting that AI assessment may provide more accurate measurement of underlying speaking ability, uncoupled from anxiety management capabilities.

Therefore, claims about AI assessment “improving” performance would be overstated and potentially misleading. Rather, the evidence suggests that AI facilitation improves assessment equity and validity by reducing construct-irrelevant anxiety effects, allowing learners to demonstrate their true speaking competence regardless of anxiety levels. This distinction between performance improvement and validity improvement is crucial for appropriate interpretation and application of these findings.

### 4.4 Digital literacy and AI-facilitated assessment

The significant positive correlation between digital literacy and relative performance in AI-facilitated assessment underscores the importance of technological competence in educational technology contexts. Students with higher digital literacy appeared better equipped to navigate and leverage the AI assessment environment, resulting in comparative performance advantages relative to human-facilitated assessment.

This finding aligns with previous research demonstrating that digital literacy enhances engagement with and benefits from educational technologies (Hillmayr et al., 2020; Zamista and Azmi, 2023). However, our study specifically highlights the relevance of digital literacy in assessment contexts, where technological proficiency may influence not only learning but also the demonstration and evaluation of knowledge and skills.

**Figure 2:**
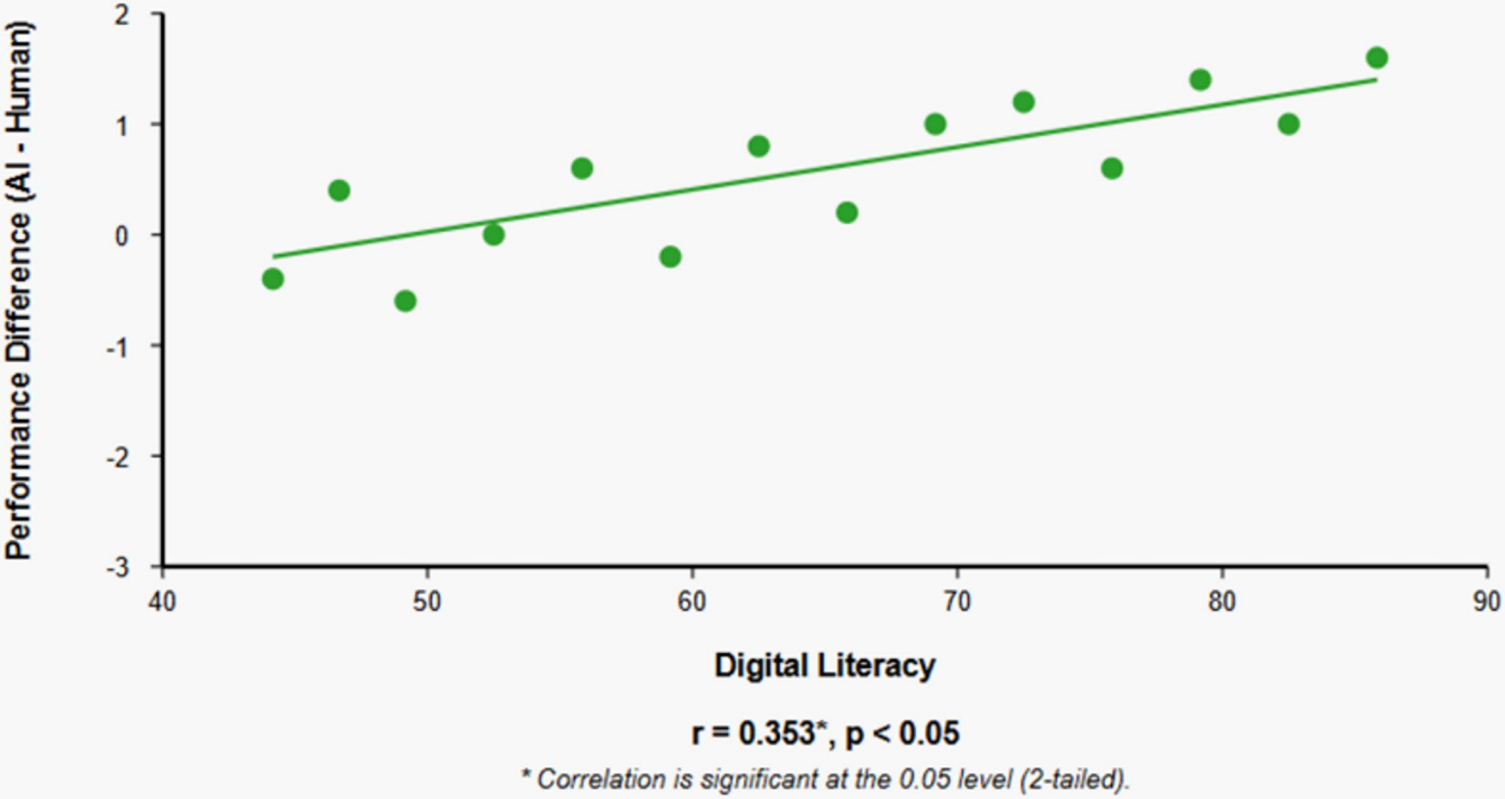
Relationship between digital literacy and relative performance in AI-facilitated assessment.

Figure 2 illustrates how higher digital literacy correlates with better relative performance in AI-facilitated assessment compared to human-facilitated assessment. The positive slope indicates that as digital literacy increases, the performance advantage in AI-facilitated assessment tends to increase as well.

The digital literacy effect raises important equity considerations for AI-facilitated assessment implementation. If technological competence influences assessment outcomes independently of language ability, AI-facilitated approaches may advantage digitally literate students while disadvantaging those with limited technological experience, potentially introducing construct-irrelevant variance into assessment results. This concern is particularly relevant in contexts with digital divides based on socioeconomic, geographical, or demographic factors.

To address this potential inequity, educational institutions implementing AI-facilitated assessments should consider providing digital literacy training as part of assessment preparation, ensuring that all students possess the technological competencies necessary to engage effectively with assessment technologies. Additionally, assessment designers should prioritize user-friendly interfaces that minimize technical complexity and cognitive load unrelated to the target language skills.

Mediation Analysis Considerations: While the current study documents a significant relationship between digital literacy and relative performance in AI-facilitated assessment (*r* = 0.353, *p* < 0.05), a formal mediation analysis would provide deeper insights into the mechanisms through which digital literacy exerts its influence. Specifically, such an analysis could test whether digital literacy mediates the relationship between assessment modality and performance, and whether digital literacy moderates the anxiety-performance relationship differently across assessment conditions.

A proposed mediation model would examine whether digital literacy affects: (1) the ease of chatbot interface navigation, thereby reducing extraneous cognitive load; (2) confidence in interacting with AI systems, thereby attenuating technology-related anxiety; and (3) the ability to leverage AI feedback features effectively, thereby enhancing metacognitive engagement with assessment results. Additionally, moderation analysis could reveal whether the anxiety-reducing effects of AI assessment are stronger for digitally literate learners or whether these effects transcend technological competence.

Such analyses require larger sample sizes than the current study provides (*n* = 48) to achieve adequate statistical power for detecting mediation effects (Fritz and MacKinnon, 2007). Future research with samples of 150+ participants would enable robust mediation and moderation modeling using structural equation approaches, potentially revealing that digital literacy’s influence operates through multiple indirect pathways rather than a single direct effect. These analyses would have significant practical implications for preparing learners to benefit equitably from AI-mediated assessments.

### 4.5 Student perceptions and experiences

The qualitative findings reveal nuanced and sometimes contradictory perceptions of AI-facilitated versus human-facilitated assessment. While quantitative data demonstrated reduced anxiety and neutralized anxiety-performance relationships in AI-facilitated assessment, most participants expressed preference for human facilitation, citing empathetic understanding and nonverbal communication as key advantages of this approach.

This apparent contradiction can be understood through the lens of Sociocultural Theory (Vygotsky, 1978), which emphasizes the fundamentally social nature of learning and assessment. Human facilitation provides social presence and interpersonal connection that many learners value despite the increased evaluative pressure and anxiety it may entail. This paradox—lower anxiety and neutralized anxiety-performance effects yet preference for human facilitation—can be understood through sociocultural and socio-emotional frameworks that emphasize the fundamentally social nature of assessment as a communicative and relational practice (Vygotsky, 1978).

Drawing on sociocultural theory, assessment is not merely a measurement event but a socially situated interaction embedded in relationships, expectations, and interpersonal dynamics (Lantolf and Thorne, 2006). Human examiners provide social presence, emotional attunement, and relational connection that transcend the purely evaluative function. Students may value these social affordances even when they simultaneously trigger evaluative anxiety, because human interaction fulfills fundamental needs for recognition, validation, and belonging that AI systems cannot address.

Research on emotional presence in online learning environments (Cleveland-Innes and Campbell, 2012; Bolender et al., 2023) demonstrates that learners distinguish between cognitive effectiveness and affective satisfaction, often preferring contexts that provide emotional connection despite higher stress. In assessment specifically, students may accept anxiety as an inherent aspect of authentic evaluation while rejecting assessment experiences that feel impersonal or mechanistic, regardless of their anxiety-reducing properties.

Additionally, the novelty of AI assessment likely contributed to this paradox. Participants’ unfamiliarity with chatbot interactions may have intensified their desire for familiar human assessment, with preference reflecting comfort-seeking rather than reasoned judgment about assessment quality. Longitudinal research examining whether preferences shift after repeated AI assessment exposure would clarify whether this paradox represents a stable phenomenon or a transitional response to technological unfamiliarity.

The finding that most participants would implement AI technologies for practice rather than assessment in their future teaching aligns with the Technology Acceptance Model (Davis, 1989), suggesting that perceived usefulness shapes technology adoption intentions.

### 4.6 Theoretical implications

This study contributes to theoretical understanding of technology-mediated assessment in several ways. First, it extends Foreign Language Anxiety Theory (Horwitz et al., 1986) by demonstrating that assessment modality (AI versus human) can significantly influence anxiety levels and, more importantly, the relationship between anxiety and performance. This suggests that anxiety is not simply an intrinsic learner characteristic but rather emerges from the interaction between individual predispositions and assessment environment characteristics.

Second, the findings enrich Cognitive Load Theory (Sweller, 1988) by illustrating how assessment modality may affect cognitive resource allocation in language production contexts. The apparent ability of AI facilitation to neutralize anxiety effects on performance suggests that this approach may reduce extraneous cognitive load associated with social monitoring and impression management, potentially enabling more cognitive resources to be devoted to the target language task.

Finally, the findings underscore the relevance of Sociocultural Theory (Vygotsky, 1978) in technology-enhanced assessment, revealing that despite objective advantages in anxiety reduction, many learners still value the social and interpersonal dimensions of human-facilitated assessment. This highlights the need for assessment theories that integrate cognitive, affective, and social perspectives to fully capture the complex dynamics of assessment experiences and outcomes.

**Figure 3:**
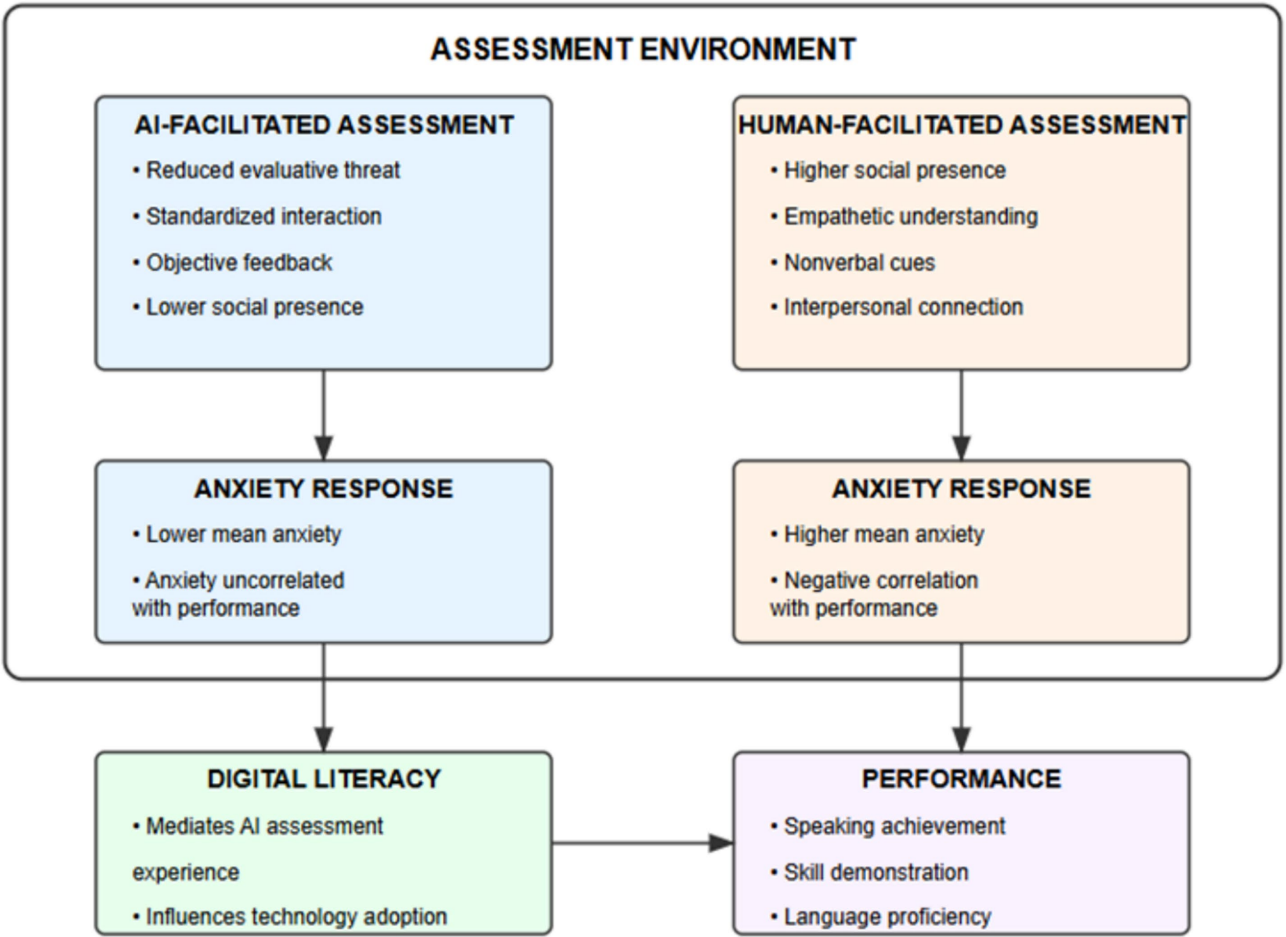
Theoretical framework for understanding AI vs. human-facilitated assessment dynamics.

Figure 3 illustrates the interrelationships among key theoretical constructs in the study. Assessment environment characteristics influence anxiety responses, which in turn affect performance outcomes.

### 4.7 Practical implications

The findings of this study have several practical implications for language education and assessment practice. First, the anxiety-reducing effect of AI facilitation suggests that AI chatbots could serve as valuable tools for creating lower-stress assessment environments, particularly for highly anxious learners who may be disadvantaged in traditional assessment formats. Implementing AI-facilitated speaking assessments as an alternative or complementary approach could enhance assessment equity by reducing the performance-inhibiting effects of anxiety.

Second, the observed relationship between digital literacy and AI-facilitated performance underscores the importance of developing students’ technological competencies alongside their language skills. Language programs should consider incorporating digital literacy development into their curricula, ensuring that students possess the technological proficiency necessary to benefit from AI-enhanced learning and assessment environments.

Third, the positive evaluation of AI feedback suggests that AI chatbots could serve as valuable tools for formative assessment and practice, even if human facilitation remains preferred for summative assessment. Implementing AI chatbots for regular speaking practice could provide students with frequent, detailed feedback without placing excessive demands on instructor time, potentially accelerating skill development through increased practice opportunities.

Finally, the finding that most participants would implement AI technologies in their future teaching practices suggests that teacher education programs should prepare students to effectively integrate AI tools into language instruction. This preparation should include not only technical skills but also pedagogical knowledge about appropriate applications and limitations of AI in various instructional contexts.

This implementation framework synthesizes findings from the current study with broader research on educational technology integration, emphasizing that successful AI chatbot deployment requires coordinated attention to multiple domains: pedagogical design (task selection, assessment alignment), technological infrastructure (interface usability, reliability), professional development (teacher training, implementation support), and student preparation (digital literacy development, orientation protocols). The model acknowledges that AI chatbots serve different functions across formative and summative assessment contexts, with greater emphasis on practice applications and feedback provision rather than high-stakes evaluation.

**Figure 4:**
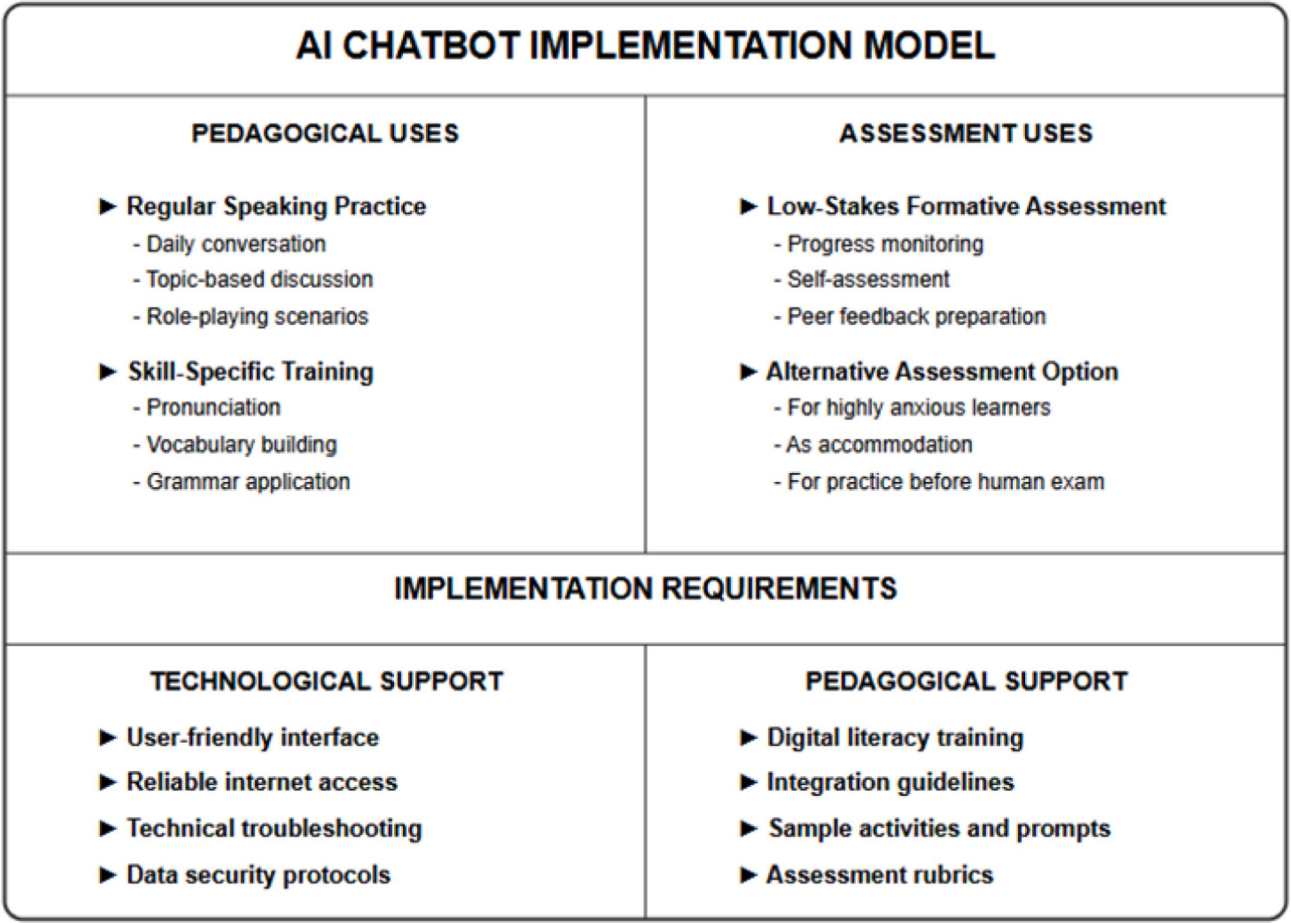
Recommended implementation model for AI chatbots in language education.

Figure 4 presents a comprehensive model for implementing AI chatbots in language education contexts, addressing both pedagogical and assessment applications along with necessary implementation support.

### 4.8 Limitations and future research directions

Several limitations of this study should be acknowledged when interpreting its findings. First, the sample size (*N* = 48) and convenience sampling approach limit the generalizability of results to broader populations. Future research should employ larger, more diverse samples and random sampling techniques to enhance external validity.

Generalizability Constraints: The study’s conduct within a single Turkish public university with English Language Teaching majors substantially limits the generalizability of findings to other populations and contexts. Turkish educational culture, characterized by relatively hierarchical teacher-student relationships and strong emphasis on formal examinations (Öğülmüş, 1991), may influence both baseline anxiety levels and responses to AI versus human assessment in ways that differ from contexts with less formal assessment traditions or more egalitarian educational relationships.

Furthermore, ELT majors represent a specialized population with stronger metalinguistic awareness, higher L2 motivation, and potentially different anxiety profiles compared to general English learners or students studying English for specific purposes. The extent to which anxiety-reduction effects observed among this population transfer to engineering students, business majors, or community language learners remains an empirical question requiring multi-site, cross-disciplinary replication.

Cultural variation in technology attitudes presents additional generalizability concerns. Research documents substantial cross-national differences in AI acceptance, trust in automated systems, and comfort with technology-mediated social interactions (Huang et al., 2019). The anxiety-reducing effects of AI assessment observed in this Turkish sample may not replicate in contexts where AI technologies evoke greater skepticism, privacy concerns, or resistance to technological replacement of human roles.

To address these generalizability limitations, we recommend: (1) Multi-site replications across diverse institutional types (community colleges, private universities, vocational schools) and geographical regions; (2) Cross-cultural comparative studies examining AI assessment effects across societies with varying technology attitudes and educational traditions; (3) Investigations with diverse learner populations including non-language-majors, adult learners, and heritage language speakers; and (4) Longitudinal studies examining whether AI assessment effects persist, amplify, or attenuate with repeated exposure and technological maturation.

Second, the study examined only short-term effects of AI facilitation on anxiety and performance. Longitudinal research is needed to determine whether anxiety reduction effects persist over time or diminish with increased familiarity with AI assessment technologies.

Looking beyond these limitations, several promising directions for future research emerge from this study. Exploring the potential of hybrid assessment approaches that combine AI and human facilitation could identify optimal integration strategies that maximize the benefits of both modalities. Investigating the development of emotionally intelligent AI systems capable of recognizing and responding to learner affective states could address the current limitations of AI in providing empathetic assessment experiences.

## 5 Conclusion

This study investigated the comparative impact of AI-facilitated and human-facilitated speaking assessments on foreign language speaking anxiety and achievement among ELT students in Turkey. The findings reveal that AI facilitation significantly reduced speaking anxiety compared to human facilitation and, more importantly, neutralized the negative relationship between anxiety and performance that characterized human-facilitated assessment. These results suggest that AI chatbots may create more equitable assessment environments, particularly for anxious learners who are disadvantaged in traditional assessment formats.

The significant relationship between digital literacy and relative performance in AI-facilitated assessment underscores the importance of technological competence in technology-enhanced educational contexts. This finding highlights the need for digital literacy development alongside language skills to ensure that all learners can effectively engage with and benefit from emerging educational technologies.

Qualitative insights from participant interviews revealed complex and sometimes contradictory perspectives on AI-facilitated versus human-facilitated assessment. While participants valued the precision, objectivity, and permanence of AI feedback, most expressed preference for human facilitation due to its empathetic qualities and interpersonal connection. This apparent contradiction suggests that optimal assessment approaches may combine elements of both modalities to leverage their complementary strengths.

In response to our research questions, we conclude that: (1) speaking anxiety is significantly lower in AI-facilitated examinations compared to human-facilitated examinations; (2) speaking anxiety negatively correlates with achievement in human-facilitated examinations but shows no significant relationship in AI-facilitated examinations; (3) digital literacy positively correlates with relative performance in AI-facilitated examinations; and (4) students perceive AI-facilitated assessment as providing more precise and objective feedback but generally prefer human facilitation for its emotional understanding and interpersonal qualities.

These findings contribute to theoretical understanding of technology-mediated assessment and offer practical guidance for implementing AI technologies in language education contexts. By illuminating the complex dynamics between assessment modality, speaking anxiety, digital literacy, and achievement, this study provides valuable insights for creating more effective and equitable assessment environments that optimize learning outcomes and experiences for diverse language learners.

## Data Availability

The raw data supporting the conclusions of this article will be made available by the authors, without undue reservation.

## Appendix A: Technical specifications of AI chatbot system

### System architecture and development

The AI chatbot system employed in this study was built upon the GPT-4 large language model architecture (OpenAI, 2023), specifically the “gpt-4-turbo-preview” version. The base model was fine-tuned using a specialized corpus comprising 15,000 spoken language samples representing CEFR proficiency levels A2 through C1, collected from authentic EFL speaking assessments conducted at multiple Turkish universities.

The system architecture incorporated three primary components: (1) an automatic speech recognition (ASR) module using Whisper AI (OpenAI, 2023) for transcribing participant speech with 94.3% word-level accuracy; (2) a natural language understanding module for analyzing linguistic features including grammatical accuracy, lexical sophistication, discourse coherence, and pronunciation quality; and (3) a dialogue management system for generating contextually appropriate prompts and follow-up questions.

### Scoring algorithm and validation

The automated scoring algorithm evaluated four dimensions aligned with the TOEFL iBT Speaking rubric: delivery, language use, topic development, and overall intelligibility. Each dimension received a preliminary score on a four-point scale based on weighted linguistic features extracted from participant responses. Algorithm validity was established through comparison with expert human ratings on 500 speaking samples, revealing strong correlations: delivery (*r* = 0.82), language use (*r* = 0.79), topic development (*r* = 0.76), and overall intelligibility (*r* = 0.85), all *p* < 0.001.

Given current limitations of fully automated assessment in evaluating pragmatic competence and creative language use (Zechner et al., 2023), we implemented a hybrid evaluation approach wherein AI-generated preliminary scores were reviewed by experienced language assessment specialists. This human oversight ensured assessment validity while retaining the anxiety-reduction benefits of AI interaction.

### Technical implementation

The chatbot interface was developed using React.js (frontend) and Python FastAPI (backend), deployed on secure university servers. Audio capture utilized Web Audio API with 48 kHz sampling rate and 16-bit depth. The user interface featured minimalist design with visual indicators (animated waveforms, color-coded status messages) providing real-time feedback about recording status. The system included automated audio checks before assessment, ensuring proper microphone configuration.

All participant interactions were encrypted and stored on secure servers complying with GDPR and institutional data protection policies. Participants provided informed consent addressing AI system data collection. Audio recordings were retained only for assessment and research purposes with scheduled deletion 12 months post-study.
